# Predictors of SARS-CoV-2 Infection and Severe and Lethal COVID-19 after Three Years of Follow-Up: A Population-Wide Study

**DOI:** 10.3390/v15091794

**Published:** 2023-08-24

**Authors:** Maria Elena Flacco, Cecilia Acuti Martellucci, Graziella Soldato, Giuseppe Di Martino, Annalisa Rosso, Roberto Carota, Marco De Benedictis, Graziano Di Marco, Rossano Di Luzio, Matteo Ricci, Antonio Caponetti, Davide Gori, Lamberto Manzoli

**Affiliations:** 1 Department of Environmental and Preventive Sciences, University of Ferrara, 44121 Ferrara, Italy; mariaelena.flacco@unife.it (M.E.F.); cecilia.martellucci@unife.it (C.A.M.); annalisa.rosso@unife.it (A.R.); 2 Local Health Unit of Pescara, 65124 Pescara, Italy; graziella.soldato@ausl.pe.it (G.S.); giuseppe.dimartino@ausl.pe.it (G.D.M.); roberto.carota@ausl.pe.it (R.C.); marco.debenedictis@ausl.pe.it (M.D.B.); graziano.dimarco@ausl.pe.it (G.D.M.); rossano.diluzio@ausl.pe.it (R.D.L.); antonio.caponetti@ausl.pe.it (A.C.); 3 Department of Medical and Surgical Sciences, University of Bologna, 40100 Bologna, Italy; matteo.ricci18@studio.unibo.it (M.R.); davide.gori4@unibo.it (D.G.)

**Keywords:** SARS-CoV-2, COVID-19, predictors, risk factors, cohort study, Italy

## Abstract

In this cohort study, the general population of an Italian Province was followed for three years after the start of the pandemic, in order to identify the predictors of SARS-CoV-2 infection and severe or lethal COVID-19. All the National Healthcare System information on biographical records, vaccinations, SARS-CoV-2 swabs, COVID-19 cases, hospitalizations and co-pay exemptions were extracted from 25 February 2020 to 15 February 2023. Cox proportional hazard analysis was used to compute the relative hazards of infection and severe or lethal COVID-19, adjusting for age, gender, vaccine status, hypertension, diabetes, major cardiovascular diseases (CVD), chronic obstructive pulmonary disease (COPD), kidney disease or cancer. Among the 300,079 residents or domiciled citizens, 41.5% had ≥1 positive swabs during the follow-up (which lasted a mean of 932 days). A total of 3.67% of the infected individuals experienced severe COVID-19 (*n* = 4574) and 1.76% died (*n* = 2190). Females, the elderly and subjects with diabetes, CVD, COPD, kidney disease and cancer showed a significantly higher risk of SARS-CoV-2 infection. The likelihood of severe or lethal COVID-19 was >90% lower among the youngest, and all comorbidities were independently associated with a higher risk (ranging from +28% to +214%) of both outcomes. Two years after the start of the immunization campaign, the individuals who received ≥2 doses of COVID-19 vaccines still showed a significantly lower likelihood of severe or lethal disease, with the lowest risk observed among subjects who received at least one booster dose.

## 1. Introduction

Since the beginning of the SARS-CoV-2 pandemic, several studies showed that the likelihood of infection and/or COVID-19 disease varied largely according to some individual characteristics, including age, gender and body mass index, and a few underlying comorbidities [1,2,3,4,5,6,7,8,9,10,11,12]. Most of these studies, however, were performed in the first phases of the pandemic, before or at the beginning of the immunization campaign [2,5,6,7], and selectively included either severely ill hospitalized patients or mildly infected people in community isolation [8,11]. However, three years after the start of the pandemic, the scenario has been deeply affected by the onset of novel viral variants and the worldwide immunization campaign [13,14,15]. In fact, very few studies include recent data—up to the first half of 2022—and little is still known about the vulnerability to SARS-CoV-2 infection and severe or lethal COVID-19 within an unselected population, largely immunized with three, or more, vaccine doses [4,10].

As the identification of high-risk clinical characteristics is crucial to facilitate early prediction, diagnosis and efficient treatment of patients [7], we performed a retrospective cohort study in the general population of an Italian Province, followed for three years after the start of the pandemic, to identify the demographic and clinical characteristics associated with an increased risk of SARS-CoV-2 infection and severe or lethal COVID-19.

## 2. Materials and Methods

This retrospective cohort study originates from previous studies evaluating COVID-19 vaccine effectiveness or SARS-CoV-2 reinfection rates on the same population and expands the follow-up up to three years after the start of the pandemic [13,16,17,18,19]. We included all the subjects residing or domiciled in the Italian Province of Pescara on 25 February 2020 (the start of the pandemic), aged 10 years or more, and followed them up to 15 February 2023.

We extracted all the information of the following official National Healthcare System datasets, which are routinely collected, updated daily and sent to the Italian Institute of Health [20]:

- Demographic data (Italian “Anagrafica”), which contained all death records;
- SARS-CoV-2 laboratory or pharmacy tests, containing the information on all positive nasopharyngeal swabs detected through RT-PCR by the regional accredited laboratories (throughout the follow-up) or through rapid antigen test by local pharmacies (since January 2021), from the start of the pandemic;
- COVID-19 vaccinations (from 1 January 2021, the start of the vaccination campaign, to 31 December 2022);
- COVID-19 database, which includes all data on officially recorded COVID-19 cases, hospitalized or not;
- Co-pay exemption dataset (Italian “Esenzioni Ticket”);
- Administrative discharge abstracts of the last ten years.

The latter three datasets were used to extract information on selected comorbidities. As an example, in the hospital discharge abstract, the following ICD-9-CM codes in any diagnosis field were extracted: 250.xx (to identify subjects with diabetes); 401.xx–405.xx (hypertension); 410.xx–412.xx, 414.xx–415.xx, 428.xx or 433.xx–436.xx (CVD); 491.xx–493.xx (COPD); 580.xx–589.xx (kidney disease); and 140.xx–172.xx or 174.xx–208.xx (cancer).

All the information from the above databases was merged through an encrypted fiscal code, using a deterministic linkage (high-quality National Tax Registry was used for the fiscal code generation and input in the main above databases).

The main outcomes were:

(a) SARS-CoV-2 infection—asymptomatic infection or mild disease, defined as fever or malaise plus at least one of the followings: sore throat, muscle pain, shortness of breath, dry cough, headache, conjunctivitis and diarrhea [21], with no hospital admission;
(b) Severe COVID-19 disease—virologically confirmed COVID-19 syndrome, diagnosed by a specialist physician and requiring hospital admission;
(c) COVID-19-related death—severe COVID-19 disease causing death within 60 days [22].

The denominator for the computation of the rates of the outcome “SARS-CoV-2 infection” was the entire population, while the denominators of “severe COVID-19” and “COVID-19-related deaths” were the population of infected individuals. Subjects were classified as “vaccinated” if they received ≥1 dose of BNT162b2, ChAdOx1 nCoV-19, mRNA-1273, NVX-CoV2373 or JNJ-78436735 vaccine, ≥14 days before the infection.

Univariate analyses compared the frequency of each outcome by age class (0–29 y, 30–59 y, 60+ y), gender, comorbidities and vaccination status (0, 1–2, 3 or more doses). The age classes were chosen to be consistent with the reports of the Italian Institute of Health [23] and with the Italian Government [24], which identified subjects aged ≥60 years as priority targets for immunization.

Cox proportional hazard analysis was then used to compute the relative hazards of infection, severe COVID-19 and COVID-19-related death after adjusting for age, gender, vaccine status and each recorded comorbidity, all included a priori. The follow-up started on 25 February 2020 and ended the day of each outcome occurrence, or was limited to 15 February 2023 for those who had no outcomes. The vaccination status (and, consequently, number of subjects in each vaccination group) varied according to the outcome, depending on the date in which the outcome occurred (before or after each vaccine dose). In example, if a subject who received three vaccine doses had a positive SARS-CoV-2 swab after the second dose, and before the third dose, he/she was included in the group “two doses only” for the analyses of the outcome “infection”, while he/she was included in the group “three or four doses” for the outcome “COVID-19-related death”. The main analyses were also repeated restricting the follow-up to the Omicron predominance period (approximately from 1 January 2022 to the end of follow-up). In these analyses, the subjects who died or were infected with SARS-CoV-2 before 1 January 2022 were excluded, and the follow-up started on that date for all the participants.

A minimum events-to-variable ratio of 10 was maintained in all multivariable models, and a Schoenfeld’s test was used to assess the validity of proportional hazards’ assumption. A two-sided *p*-value < 0.05 was considered significant. Stata, version 13.1 (Stata Corp., College Station, TX, USA, 2014), was used for all analyses.

## 3. Results

Overall, a total of 300,079 residents or domiciled in the Province of Pescara were included in the study and followed up to 1086 days (932 on average). The baseline characteristics of the sample are reported in Table 1: a total of 48.8% were males; 31.7% were aged 60 year or more at the start of the study; 14.1% were diagnosed with hypertension; and 8.5% had a previous major cardiovascular disease (stroke or myocardial infarction). Most of the population received three or more COVID-19 vaccine doses (62.2%), while 16.7% were unvaccinated.

During the follow-up, 41.5% had at least one SARS-CoV-2 infection (*n* = 124,622). Among the infected, the incidence of severe COVID-19 was 3.67%, while the proportion of COVID-19-related deaths was 1.76% (Table 1).

### 3.1. Predictors of SARS-CoV-2 Infection

As shown in Table 2, the proportion of infection significantly differed according to all of the included predictors, with the larger differences observed by age (34.3% among the elderly versus ≈ 45% in the other age classes), COPD (46.8% versus 41.3% in the rest of the population) and vaccination status (44.5% among the unvaccinated versus 35.7% among those who received three or more doses, or 60.0% among the recipients of two doses only). In the multivariate analysis (Table 3), males showed a lower likelihood of infection, whereas a younger age, diabetes, CVD, COPD, kidney disease and cancer significantly increased the risk of SARS-CoV-2 infection (all *p* < 0.001). Concerning vaccination status, as compared to the unvaccinated, the probability of being infected was significantly lower among those who received one or three or more vaccine doses, while it was higher in the group of subjects who received two doses only. When the analyses were repeated restricting the follow-up to the Omicron predominance period (Table S1), the multivariable results were concordant with those of the overall sample, with the notable exception of an increased risk of infection for all vaccinated subjects.

### 3.2. Predictors of Severe COVID-19 and COVID-19-Related Death

The incidence of both outcomes significantly and substantially varied by age, all recorded comorbidities and vaccination status (Table 2). While the proportion of severe COVID-19 or COVID-19-related deaths were as low as 0.30% and 0.03%, respectively, among the individuals aged less than 30 years, the values increased to 9.82% and 6.20%, respectively, among the subjects aged 60 or more years. Similarly, among the subjects with hypertension, diabetes, CVD, COPD, kidney disease or cancer, the incidence of severe COVID-19 and COVID-19-related death were at least three and five times higher, respectively. Finally, the unvaccinated individuals showed much higher rates of both COVID-19 (10.8%) and death (7.57%) when compared to the vaccinated subjects. Among the latter, the lowest incidence of both outcomes was observed among the individuals who received at least two vaccine doses.

**Figure 1:**
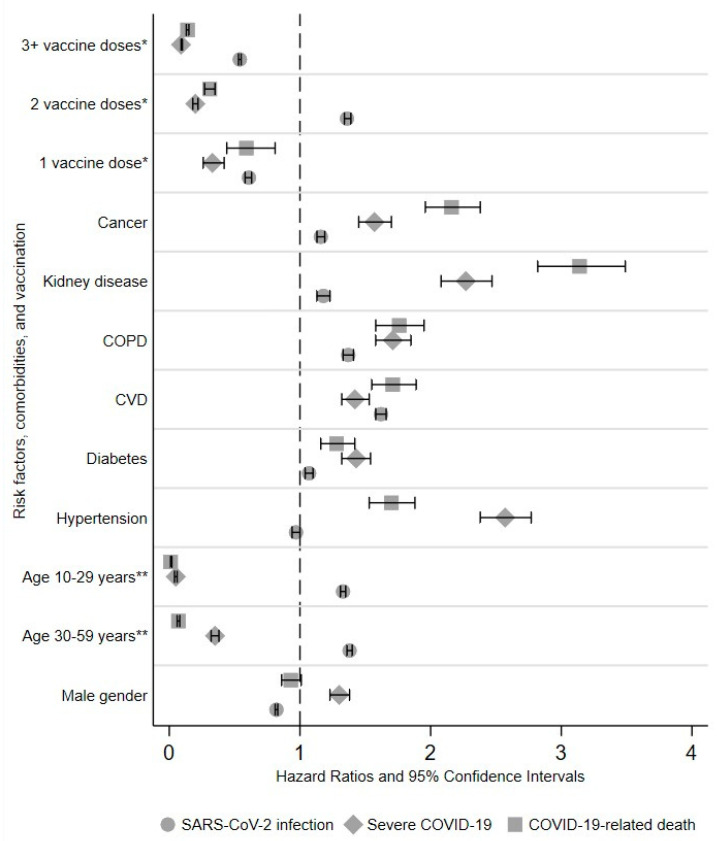
Forest plot displaying the adjusted hazards ratios of SARS-CoV-2 infection and severe and lethal COVID-19, by the selected predictors. Values higher than 1 indicate a higher risk of infection or disease of the subjects with the specific characteristic—e.g., males, as compared to the rest of the population—e.g., females. The horizontal bars represent the 95% confidence intervals. * Reference category: unvaccinated subjects. ** Reference category: Individuals aged 60 years or more.

In the multivariable analyses (Table 3 and Figure 1), as compared to the elderly, the likelihood of COVID-19 or COVID-19-related death were >90% lower among the youngest, and the presence of all comorbidities was independently associated with a higher (ranging from +28% to +214%) risk of both outcomes. Compared to the unvaccinated, the subjects who received any dose of vaccine showed a substantially lower likelihood of both severe COVID-19 and COVID-19-related death, with a clear dose–response trend (for COVID-19: HR = 0.33 for those who received one dose only; HR = 0.20 after two doses only; HR = 0.09 for those who received at least one booster dose). Finally, a positive association was observed between male gender and severe COVID-19 only (HR = 1.30).

During the Omicron predominance period, the overall findings were very similar to those of the overall follow-up (Table S1), with the partial exceptions of a generally lower protection for the vaccinated subjects (and no protection for those who received a single dose), and a larger difference in the risk of severe or lethal COVID-19 between the elderly and the adults, whose death rate dropped.

## 4. Discussion

The main findings of this population-based cohort study are the following: First, a younger age, female gender and diabetes, cancer and cardiovascular, respiratory or kidney diseases were all independent predictors of SARS-CoV-2 infection. Second, it is confirmed that, among SARS-CoV-2-positive subjects, an older age, male gender and a history of hypertension, diabetes, CVD, COPD, kidney disease or cancer were associated with a higher likelihood of developing a severe or lethal COVID-19 disease. In particular, the subjects with at least one among the recorded comorbidities showed a three to five times higher likelihood of disease progression, as compared to those without any of the above conditions. Third, as compared to unvaccinated individuals, adjusting for age, gender and for all the recorded comorbidities, those who received any dose of the SARS-CoV-2 vaccine showed a substantially lower risk of both severe and lethal COVID-19, with a clear dose–response trend for both outcomes. Finally, the individuals who received one or more booster doses showed an approximate 50% reduction in the risk of SARS-CoV-2 infection, but a higher rate of infections was observed among the subjects who received only two vaccine doses, in line with previous findings on the same population [25]. Notably, however, when the analyses were restricted to the Omicron predominance period, only the subjects who received two or more vaccine doses showed a significantly lower risk of severe or lethal COVID-19, and vaccination did not protect from infection.

The role of age as a very strong and independent predictor of severe or lethal COVID-19 is well known since the start of the pandemic [1,3,4,5,6,7,8,9,10,11,12,26]. Consistent with previous estimates, the rates of lethal COVID-19 among the elderly were very high (approximately 1 out of 16 died) and 206 times higher than those observed among the young. To date, the causal pathways linking age and severe or lethal disease are not entirely disclosed [27]. A recent study suggested that ageing is associated with the elevation of the biomarkers that promote endothelium and coagulation system activation, inflammation and organ damage [28]. In any case, it remains extremely complex to dissect the specific role of age, age-related risk factors (such as chronic comorbidities), host response and severe or lethal COVID-19 [28,29]. The present analysis, adjusting for comorbidities or other known predictors, allowed us to explicitly “factor out” their effect, adding new data to quantify the isolated role of age [27].

Age was also independently, inversely associated with the risk of infection: higher rates were observed among younger or adult individuals (<60 years), and the association remained stable after disentangling the role of the other predictors. Different behaviors across age-classes are consistently identified as the possible underlying explanation of the phenomenon [13,30], and prior evidence from the same cohort showed a decreasing trend of infections with increasing age, irrespective of vaccination status and number of vaccine doses [13,25]. However, some studies from both European and non-European countries suggested that the risk of SARS-CoV-2 infection is around 30% lower among adolescents (10–20 years) as compared to older ages [30,31]. Thus, there is still no consensus in the available literature on the topic.

We observed higher rates of infection among females, whereas males showed a higher risk of severe COVID-19, in line with most of the available literature on the topic [1,2,7,11,12,26]. The potential explanations for such a peculiar finding are still uncertain [1,2,7,26]: First, prior studies have suggested potential differences between genders in health-seeking behaviors—a lower attitude to routine diagnostic testing among males—or in occupational factors—a higher work-related exposure to the contagion of females [19], which might explain, at least in part, the higher risk of infection among females. Second, it has been suggested that the observed association between male gender and severe COVID-19 might be partly due to the unmeasured confounding effect of some comorbidities, which are more frequent among men [2]. Finally, the gender difference might be a consequence of the dissimilar immune response, with males showing higher circulating concentration of Angiotensin-Converting Enzyme II (ACE2) [32], leading to increased viral binding to ACE2, higher levels of Angiotensin II and consequent pulmonary vasoconstriction, inflammation and oxidative organ damage, ultimately promoting higher risk of acute lung injury [3].

After adjusting for age, gender and vaccination status, our findings show a significant increase in the likelihood of SARS-CoV-2 infection, severe COVID-19 and COVID-19-related death among the subjects with a history of diabetes, CVD, COPD, kidney disease and cancer. In particular, we observed the highest hazard ratios of severe or lethal COVID-19 among individuals with kidney disease. In line with our findings, a meta-analysis, including age- and gender-adjusted data from 88 studies and more than 6 million SARS-CoV-2-positive subjects, identified COPD, CVD, hypertension, diabetes, cancer and chronic renal disease as the conditions more closely linked to COVID-19-related death [12]. Additionally, an umbrella meta-analysis summarizing the results of 16 previous meta-analyses (although none of these published after 2021) reported the highest likelihood of severe COVID-19 among subjects with cardiovascular or chronic kidney diseases [2]. Indeed, it has been suggested that these two conditions share some characteristics, including chronic pro-inflammatory states and innate or adaptive immunity dysfunction, which might facilitate a dysregulated immune response against SARS-CoV-2 [33]. Thus, the present findings confirm and expand the current evidence needed to establish the priorities for those requiring booster vaccine doses [7] or to assist triage decision-making for asymptomatic or infected individuals [2,8].

Beyond pre-existing clinical conditions and demographic factors, the present results expand the current evidence on the effectiveness of COVID-19 vaccines in preventing the progression of SARS-CoV-2 infection towards severe or lethal disease [13,18,34]. Notably, although a significantly lower risk of disease was observed after any dose, the likelihood of both severe and lethal COVID-19 decreased by more than 85% among the subjects who received at least one booster dose. Although vaccination effectiveness was generally lower during the Omicron predominance period, the subjects who received at least a booster dose still showed a 70% lower risk of severe or lethal COVID-19. During the Omicron wave, however, all vaccinated subjects showed a higher risk of infection. This finding should be interpreted with caution, since it was likely caused by the restrictive measures adopted in Italy to control the epidemic, which allowed a much higher mobility to vaccinated individuals, who were thus more likely to be exposed to contagion than the unvaccinated [35,36]. Overall, these findings are in line with several previous studies [37,38,39,40] and provide novel data on the duration of the protection conferred by booster doses after more than a year from the start of the booster doses administration.

In partial agreement with preliminary findings from the same cohort [25], we also observed a positive association between vaccination with two doses and SARS-CoV-2 infection, while a single dose, as well as one or more booster doses, were associated with a reduced risk. This counterintuitive finding, however, needs to be interpreted with caution. As previously discussed [13,18,19], it is likely a consequence of the different set of restrictive measures issued in Italy to vaccinated and unvaccinated individuals, thus leading to different behaviors by vaccination status. Indeed, for all Italian citizens, three vaccine doses, or two (in the case of a previous positive swab), were mandatory to retain their job or access a number of environments (including universities, hospitals and public places) [41]. Thus, given the much higher mobility granted to the individuals with two doses and fully recovered, these were much more likely to be exposed to contagion than the unvaccinated. Moreover, as of February 2022, a SARS-CoV-2 test was no longer mandatory for asymptomatic close contacts of a confirmed case [42], and unvaccinated individuals may have been more likely to avoid testing than vaccinated subjects, for ideological reasons or to avoid sanitary confinement [43].

In addition to the outcomes investigated in this study, several studies evaluated the predictors and severity of SARS-CoV-2 reinfections, which are also central to optimize the diagnosis treatment of a population that has already been infected [16,19,44,45]. This investigation requires different selection criteria and specific data modeling and analysis, which are currently being set. In the first preliminary analyses, in this population, we found a 5.4% rate of reinfections, which lead to 51 severe (*n* = 33) or lethal (*n* = 18) COVID-19 cases. These findings are however preliminary, and the results of the extended analyses will be presented in a following report.

In addition to providing the longest evidence to date available on the predictors of SARS-CoV-2 infection, severe and lethal COVID-19, the present study has other strengths that should be mentioned. First, it was based upon multiple official datasets collecting healthcare and demographic data, routinely updated, on the entire population of an Italian Province. Beyond the possibility of relying upon a very large, unselected sample, the use of multiple datasets enabled to trace back the clinical history of the included subjects for a decade, with a high level of accuracy in the identification of the underlying comorbidities. In addition, the possibility to adjust the analyses for multiple conditions (which may influence the risk of COVID-19 hospitalization and death) likely reduced the risk of misclassification when defining COVID-19-related deaths [13]. This study had also some limitations that must be considered when interpreting the results. First, we could not extract information on other potential predictors of severe or lethal COVID-19, such as tobacco smoking and body mass index, as they are not routinely recorded in any of the adopted datasets. Second, no data were available on the severity of the underlying comorbidities, nor on the types of medications used. Third, as already discussed, testing policies and restrictive measures applied in Italy likely influenced the results on vaccine effectiveness against infection [13,18]. Finally, our definition of infection was based upon the available laboratory information, which collected all positive swabs. Given that the monitoring system cannot detect all asymptomatic infections, the resulting infection rate is certainly underestimated [19], with consequent changes in the estimates of SARS-CoV-2 case–fatality rate. However, how this issue may affect the comparison between vaccinated and unvaccinated subjects is still unclear, as it has not been determined whether the rate of undetected infections differs by vaccination status [46].

## 5. Conclusions

In conclusion, this large population-based cohort study confirmed previous findings showing that elderly individuals, males and those with a history of hypertension, diabetes, COPD, CVD, cancer and kidney disease are at a significantly higher risk of both severe COVID-19 and COVID-19-related death. Moreover, after two years from the start of the immunization campaign, the individuals who received at least two doses of COVID-19 vaccines still showed a significantly lower risk of severe or lethal disease, with the lowest risk observed among those who received at least one booster dose.

## Figures and Tables

**Table 1:** Overall characteristics of the sample.

	Overall Sample
	(*n* = 300,079)
Male gender, % (n)	48.8 (146,435)
Mean age in years (SD)	49.1 (20.9)
Age class in years, % (n)	
- 10–29	21.5 (64,614)
- 30–59	46.8 (140,460)
- 60 or more	31.7 (95,005)
Risk factors and comorbidities, % (n) ^A^	
Hypertension	14.1 (42,420)
Diabetes	5.6 (16,674)
CVD	8.5 (25,557)
COPD	4.0 (11,912)
Kidney disease	2.1 (6297)
Cancer	5.9 (17,668)
Vaccination status, % (n)	
- Unvaccinated	16.7 (49,994)
- 1 dose ^B^	5.1 (15,409)
- 2 doses ^C^	16.0 (47,974)
- 3 or more doses ^D^	62.2 (186,702)
SARS-CoV-2 infection, % (n)	41.5 (124,622)
Severe COVID-19, % (n) (among the infected)	3.67 (4574)
COVID-19-related death, % (n) (among the infected)	1.76 (2190)
Mean follow-up in days (SD) ^E^	932 (225)

SD = standard deviation. n = number of participants. COVID-19: virologically confirmed COVID-19 disease, diagnosed by a specialist physician and requiring hospital admission. ^A^ Subjects with the selected comorbidities in the regional co-pay exemption database (Italian “Esenzioni Ticket” file), or in the regional COVID database, or with hospital admission in the last ten years (from the Italian SDO database of administrative discharge abstracts). Please see the Section 2 for more details on the ICD-9-CM codes. ^B^ Subjects who received only one dose of BNT162b2, mRNA-1273, ChAdOx1 nCoV-19 or NVX-CoV2373 vaccines between 2 January 2021 and 31 December 2022. ^C^ Subjects who received only two doses of BNT162b2, mRNA-1273, ChAdOx1 nCoV-19, or NVX-CoV2373 vaccines, or one dose of the JNJ-78436735 vaccine between 2 January 2021 and 31 December 2022. ^D^ Subjects who received three or four doses of BNT162b2, mRNA-1273, ChAdOx1 nCoV-19, JNJ-78436735 or NVX-CoV2373 vaccines between 2 January 2021 and 31 December 2022. ^E^ The end of follow-up was the date of infection or 15 February 2023. The start of follow-up was 25 February 2020.

**Table 2:** Incidence of the outcomes according to the selected predictors.

	SARS-CoV-2 Infection	Severe COVID-19 ^Ʊ^	COVID-19-Related Death ^Ʊ^
	(*n* = 124,622)	(*n* = 4574)	(*n* = 2190)
Gender	%, *	%, *	%, *
Females	43.7	3.11	1.67
Males	39.2	4.33	1.86
Mean age in years (SD)	46.6 (20.0)	68.7 (16.7)	79.1 (12.4)
Age class in years	%, *	%, *	%,*
10–29	44.8	0.30	0.03
30–59	44.9	2.04	0.25
60 or more	34.3	9.82	6.20
Risk factors and comorbidities ^A^	*	*	*
No hypertension	42.2	2.01	0.79
Hypertension	37.4	15.04	8.41
	*	*	*
No diabetes	41.7	2.96	1.32
Diabetes	38.4	16.84	9.78
	*	*	*
No CVD	41.2	2.30	0.87
CVD	44.9	17.20	10.46
	*	*	*
No COPD	41.3	3.12	1.41
COPD	46.8	15.39	9.26
	*	*	*
No kidney disease	41.6	3.10	1.32
Kidney disease	39.3	31.86	23.45
	*	*	*
No cancer	41.7	3.21	1.36
Cancer	38.7	11.52	8.59
Vaccination status ^B^			
- Unvaccinated, n/N	27,346/61,458	2963/27,346	1202/15,882
%	44.5	10.84	7.57
- 1 dose, n/N ^C^	2181/6341	68/2181	100/11.249
%	34.4	3.12	0.89
- 2 doses, n/N ^D^	30,221/50,359	483/30,221	230/27,836
%	60.0	1.60	0.83
- 3 or more doses, n/N ^E^	64,874/181,921	1060/64,874	658/69,655
%	35.7	1.63	0.94
Mean follow-up in days (SD) ^F^	749 (203)	493 (280)	616 (315)

SD = standard deviation. If not differently stated, the values in the tables are expressed as % (n), where *n* = number of participants. The proportions refer to the number of subjects with the outcome divided by the total number of subjects in the specific category. As an example, 43.7% of the females had a SARS-CoV-2 infection during the follow-up, as compared to 39.2% of the males. * Univariate chi-squared *p* < 0.001 for the differences between, e.g., females and males, or diabetics and non-diabetics. n/N = number of subjects with the outcome/Total number of subjects. Unless differently stated, the row percentages are shown. COVID-19: virologically confirmed COVID-19 disease, diagnosed by a specialist physician and requiring hospital admission. ^Ʊ^ Analyses restricted to the 124,622 infected subjects. ^A^ Subjects with the selected comorbidities in the regional co-pay exemption database (Italian “Esenzioni Ticket” file), or in the regional COVID database, or with hospital admission in the last ten years (from the Italian SDO database of administrative discharge abstracts). Please see the Section 2 for more details on the ICD-9-CM codes. ^B^ The number of subjects in each vaccine group varied according to the outcome, depending on the date in which the outcome occurred (before or after each vaccine dose). As an example, if a subject who received three vaccine doses had a positive SARS-CoV-2 swab after the second dose, but before the third dose, he/she was included in the group “two doses only” for the analyses of the outcome “infection” and in the group “three or four doses” for the outcome “death”. Overall, the number of subjects. ^C^ Please see Table 1, footnote ^B^. ^D^ Please see Table 1, footnote ^C^. ^E^ Please see Table 1, footnote ^D^. ^F^ The end of follow-up was the date of infection, COVID-19 or death, or 15 February 2023 for those who had no outcomes. The start of follow-up was 25 February 2020.

**Table 3:** Adjusted hazards ratios (HR; 95% confidence interval, CI) ^A^ of each recorded outcome.

Outcomes	SARS-CoV-2 Infection	Severe COVID-19 ^B^	COVID-19-Related Death ^B^
	HR (95% CI)	HR (95% CI)	HR (95% CI)
Male gender	0.82 (0.81–0.83) *	1.30 (1.23–1.38) *	0.93 (0.86–1.01)
Age class in years			
60 or more	1 (Ref. cat.)	1 (Ref. cat.)	1 (Ref. cat.)
30–59	1.38 (1.36–1.40) *	0.35 (0.32–0.38) *	0.07 (0.06–0.08) *
10–29	1.33 (1.31–1.35) *	0.05 (0.04–0.06) *	0.01 (0.01–0.02) *
Risk factors and comorbidities			
Hypertension	0.97 (0.94–1.00)	2.57 (2.38–2.77) *	1.70 (1.53–1.88) *
Diabetes	1.07 (1.04–1.10) *	1.43 (1.32–1.54) *	1.28 (1.16–1.42) *
CVD	1.62 (1.58–1.66) *	1.42 (1.32–1.53) *	1.71 (1.55–1.89) *
COPD	1.37 (1.33–1.41) *	1.71 (1.58–1.85) *	1.76 (1.58–1.95) *
Kidney disease	1.18 (1.13–1.23) *	2.27 (2.08–2.47) *	3.14 (2.82–3.49) *
Cancer	1.16 (1.13–1.19) *	1.57 (1.45–1.70) *	2.16 (1.96–2.38) *
Vaccination status			
- Unvaccinated	1 (Ref. cat.)	1 (Ref. cat.)	1 (Ref. cat.)
- 1 dose ^C^	0.61 (0.58–0.63) *	0.33 (0.26–0.42) *	0.59 (0.44–0.81) *
- 2 doses ^D^	1.36 (1.34–1.39) *	0.20 (0.18–0.22) *	0.31 (0.27–0.35) *
- 3 or more doses ^E^	0.54 (0.53–0.55) *	0.09 (0.09–0.10) *	0.14 (0.13–0.15) *

COVID-19 = virologically confirmed COVID-19 disease, diagnosed by a specialist physician and requiring hospital admission. ^A^ Based on Cox proportional hazards models. ^B^ Analyses restricted to the subjects who had at least one positive SARS-CoV-2 swab during the follow-up. ^C^ Please see Table 1, footnote ^B^. ^D^ Please see Table 1, footnote ^C^. ^E^ Please see Table 1, footnote ^D^ * *p* < 0.001.

## Data Availability

The data presented in this study are available upon reasonable request from the corresponding author.

## Supplementary Materials

The following supporting information can be downloaded at: https://www.mdpi.com/article/10.3390/v15091794/s1, Table S1: Adjusted hazards ratios (HR; 95% confidence interval—CI) of each recorded outcome, during the Omicron predominance (from 1 January 2022 to the end of follow-up).
