# Supplementary material for: Predictors of SARS-CoV-2 Infection and Severe and Lethal COVID-19 after Three Years of Follow-Up: A Population-Wide Study
Source: Viruses. 2023 Aug 24;15(9):1794. doi: 10.3390/v15091794 (PMC10534678; doi:10.3390/v15091794)
Supplement: Supplementary file 1 [file viruses-15-01794-s001.zip › viruses-2557754-supplementary.pdf]

Table S1. Adjusted hazards ratios (HR; 95% confidence interval - CI) [A] of each recorded outcome, during the Omicron predominance (from 1 January, 2022 to the end of follow-up) [Ω].

**Outcomes**	**SARS-CoV-2 infection** HR (95% CI)	**Severe COVID-19** [B] HR (95% CI)	**COVID-19-Related Death** [B] HR (95% CI)
Male gender	0.82 (0.81-0.83)*	0.91 (1.83-1.00)	0.83 (0.73-0.95)*
Age class in years			
60 or more	1 (Ref. cat.)	1 (Ref. cat.)	1 (Ref. cat.)
30–59	1.31 (1.29-1.34)*	0.04 (0.03-0.07)*	0.01 (0.00-0.02)*
10–29	1.40 (1.38-1.43)*	0.28 (0.24-0.32)*	0.06 (0.05-0.08)*
Risk factors and comorbidities			
Hypertension	1.00 (0.97-1.02)	2.32 (2.05-2.63)*	1.69 (1.45-1.98)*
Diabetes	1.01 (0.97-1.04)	1.34 (1.19-1.51)*	1.23 (1.06-1.44)*
CVD	1.26 (1.22-1.29)*	1.42 (1.32-1.53)*	2.12 (1.82-2.46)*
COPD	1.26 (1.22-1.30)*	1.91 (1.70-2.15)*	1.98 (1.69-2.33)*
Kidney disease	1.11 (1.06-1.17)*	2.52 (2.23-2.84)*	3.24 (2.74-3.82)*
Cancer	1.12 (1.09-1.15)*	2.11 (1.88-2.37)*	2.78 (2.42-3.20)*
Vaccination status			
- Unvaccinated	1 (Ref. cat.)	1 (Ref. cat.)	1 (Ref. cat.)
- 1 dose [C]	1.28 (1.22-1.35)*	0.76 (0.54-1.06)	1.09 (0.71-1.69)
- 2 doses [D]	3.43 (3.35-3.51)*	0.59 (0.50-0.68)*	0.75 (0.60-0.93)*
- 3 or more doses [E]	1.47 (1.44-1.50)*	0.26 (0.23-0.30)*	0.30 (0.25-0.37)*

[Ω] The 27,912 subjects who died or had a SARS-CoV-2 infection before January 1, 2022 were excluded from the sample.
COVID-19 = virologically confirmed COVID-19 disease, diagnosed by a specialist physician and requiring hospital admission.
[*] $p<0.001$.
[A] Based on Cox proportional hazards models.
[B] Analyses restricted to the subjects who had at least one positive SARS-CoV-2 swab during the follow-up.
[C] Subjects who received only one dose of BNT162b2, mRNA-1273, ChAdOx1 nCoV-19 or NVX-CoV2373 vaccines between January 2, 2021 and December 31, 2022.
[D] Subjects who received only two doses of BNT162b2, mRNA-1273, ChAdOx1 nCoV-19, or NVX-CoV2373 vaccines, or one dose of JNJ-78436735 vaccine, between January 2, 2021 and December 31, 2022.
[E] Subjects who received three or four doses of BNT162b2, mRNA-1273, ChAdOx1 nCoV-19, JNJ-78436735 or NVX-CoV2373 vaccines between January 2, 2021 and December 31, 2022.
